# Transcriptomics, NF-κB Pathway, and Their Potential Spaceflight-Related Health Consequences

**DOI:** 10.3390/ijms18061166

**Published:** 2017-05-31

**Authors:** Ye Zhang, Maria Moreno-Villanueva, Stephanie Krieger, Govindarajan T. Ramesh, Srujana Neelam, Honglu Wu

**Affiliations:** 1 NASA Kennedy Space Center, Cape Canaveral, FL 32899, USA; ye.zhang-1@nasa.gov (Y.Z.); srujana.neelam@nasa.gov (S.N.); 2 NASA Johnson Space Center, Houston, TX 77058, USA; maria.moreno-villanueva@uni-konstanz.de (M.M.-V.); stephanie.s.krieger@nasa.gov (S.K.); 3 University of Konstanz, 78464 Konstanz, Germany; 4 KBR Wyle, Houston, TX 77058, USA; 5 Norfolk State University, Norfolk, VA 23504, USA; gtramesh@nsu.edu; 6 University Space Research Association, Columbia, MD 21046, USA

**Keywords:** transcriptome, spaceflight, NF-κB pathway, human disease

## Abstract

In space, living organisms are exposed to multiple stress factors including microgravity and space radiation. For humans, these harmful environmental factors have been known to cause negative health impacts such as bone loss and immune dysfunction. Understanding the mechanisms by which spaceflight impacts human health at the molecular level is critical not only for accurately assessing the risks associated with spaceflight, but also for developing effective countermeasures. Over the years, a number of studies have been conducted under real or simulated space conditions. RNA and protein levels in cellular and animal models have been targeted in order to identify pathways affected by spaceflight. Of the many pathways responsive to the space environment, the nuclear factor kappa-light-chain-enhancer of activated B cells (NF-κB) network appears to commonly be affected across many different cell types under the true or simulated spaceflight conditions. NF-κB is of particular interest, as it is associated with many of the spaceflight-related health consequences. This review intends to summarize the transcriptomics studies that identified NF-κB as a responsive pathway to ground-based simulated microgravity or the true spaceflight condition. These studies were carried out using either human cell or animal models. In addition, the review summarizes the studies that focused specifically on NF-κB pathway in specific cell types or organ tissues as related to the known spaceflight-related health risks including immune dysfunction, bone loss, muscle atrophy, central nerve system (CNS) dysfunction, and risks associated with space radiation. Whether the NF-κB pathway is activated or inhibited in space is dependent on the cell type, but the potential health impact appeared to be always negative. It is argued that more studies on NF-κB should be conducted to fully understand this particular pathway for the benefit of crew health in space.

## 1. Introduction

Different from the surface of the Earth, humans in space experience a number of environmental stress factors. In orbits around the Earth or during transit to the Moon or Mars, the lack of gravity is one of the most significant environmental factors responsible for many physiological changes in humans. Gravity of a partial g value can also be experienced on the surface of the Moon or Mars, or artificially generated during transit to these destinations.

Apart from microgravity, astronauts are exposed to cosmic radiation during space missions [1]. Although various forms for radiation exist in space, only three sources are of major concern for astronauts’ health. In low Earth orbits (LEO), the majority of radiation comes from protons trapped in the geomagnetic field. In free space, galactic cosmic radiation (GCR) consists of high energy particles ranging from protons to extremely heavy ions [2]. Heavy ions are known to have a high linear energy transfer (LET) that can produce greater biological effects than low-LET radiation for the same absorbed dose. During transit to the Moon or Mars, or on the surface of these objects, astronauts are also in danger of exposure to protons released from large solar particle events (SPE). High energy GCR and SPE protons are able to penetrate through the geomagnetic field to reach LEO, especially in high inclination orbits [2].

How these environmental factors affect human health has been a major concern for long-duration space missions. Among the reported effects of spaceflight are bone and muscle loss due to disuse of the organs [3,4], and suppression of immune functions [5,6]. Other health risks, such as cancer induced from exposure to space radiation, have been identified for long-duration missions, although no significantly elevated cancer death among the astronauts have been reported [7].

Understanding these spaceflight-related health consequences at the molecular level is critical for accurately assessing the associated risks and for the development of effective countermeasures, especially considering the prospect that future medical treatment will be based on individual’s genetic background [8]. In recent years, a number of studies have been reported in which cultured human cells or rodents were flown to space in attempts to uncover any unique molecular pathways in response to the space environment. As opportunities for conducting experiments in space are rare, more studies have been reported using ground based analogs that simulate microgravity. The widely used microgravity analogs are rotating wall vessels (RWV, including 2D clinostats) and the random positioning machine (RPM) for cultured cells [9], and hindlimb unloading (HU) for rodents [10]. For humans, bed rest is used to simulate disuse of the bone and muscle [11]. Space radiation has been simulated on the ground using high energy particles of varying charges and energies generated using accelerators [12]. It has been known that simulated microgravity produces only some, but not all, of the biological effects of the true microgravity condition.

Among the different pathways that have been investigated in response to the space environment, the nuclear factor kappa-light-chain-enhancer of activated B cells (NF-κB) network appears to be commonly affected in many different cell types and organs of animals. As a protein complex that controls the transcription of DNA, cytokine production and cell survival, NF-κB is activated in response to external stimuli including infection, inflammation, radiation, and oxidative stress resulting in upregulation of a number of downstream stress response genes such as inducible nitric oxide synthase (*iNOS*), cyclooxygenase-2 (*COX-2*), tumor necrosis factor alpha (*TNFα*), and intracellular adhesion molecule (*ICAM*) [13,14,15]. The NF-κB family of transcription factors comprises p65 (RELA), RELB, c-REL, NF-κB1 (p50), and NF-κB2 (p52). IκB kinase (IKK) complex controls the activation of NF-κB through the canonical (classical) pathway by degrading the IκB inhibitor and releasing p65/p50 dimers to the nucleus, or through the nuclear translocation of RELB/p52 via a noncanonical (alternative) pathway [16]. Activation of NF-κB/REL protein involves phosphorylation, ubiquitination, and subsequent degradation of the inhibitory protein IκB, which in turn results in the nuclear translocation of NF-κB/REL proteins [17]. The c-REL subunit is involved in NF-κB heterodimer formation, which forms essential transcription factor complexes involved in all types of cellular processes, including cellular metabolism, and chemotaxis. Previous studies have indicated that *c-REL* gene is exclusively expressed in immune cells and plays a role in the regulation of proliferation, adhesion, survival, and immune and inflammatory responses [18]. However, more recent studies demonstrated that *c-REL* also plays a role in non-immune cells. For instance, *c-REL* deficient mice are protected from developing cardiac and skin fibrosis indicating a new role of *c-REL* in regulating cardiac remodeling and epidermal proliferation and homeostasis [19]. Phosphorylation and acetylation of RELA are crucial post-translational modifications required for NF-κB activation [20]. 

It has been well documented that alterations of the NF-κB pathway are associated with diseases, many of which are of concern for spaceflight [21]. For instance, NF-κB plays a role in osteoclastogenesis [22] and changes in osteoblast activities [23,24]. NF-κB is also suggested to play a role in muscle atrophy due to its increased expression in cardiac cell unloading [25].

This review focuses on the NF-κB pathway in response to the space environment. First, we survey omics studies that identified NF-κB as one of the major pathways in response to altered gravity conditions. Then we review the studies specifically targeting NF-κB in the organs or cell types that are associated with the known spaceflight related health risks, including immune dysfunction, bone or muscle loss, CNS dysfunction, and risks associated with space radiation exposure. Both studies conducted in space and under simulated spaceflight conditions are considered, and are summarized in Table 1. In Table 2, we summarize the studies using high energy charged particles on the ground.

## 2. Transcriptomics and the NF-κB Pathway

A number of omics studies conducted in space or using ground-based microgravity simulators have been reported in the literature. While these omics investigations did not specifically target the NF-κB pathway, several of them revealed expression changes of the genes in the NF-κB network. In TK6 human lymphoblastoid cells cultured for 72 h in RWV, the expression of both mRNA and microRNA (miRNA) was differentially regulated [26]. Pathway analysis of the genes having significant expression changes revealed activation of the NF-κB pathway in response to simulated microgravity. In addition, several miRNAs whose expression was altered may have influenced the expressions of the genes in the NF-κB network [26]. Analysis of human T cells after activation on the Soyuz 13S spaceflight mission showed 47 genes downregulated in microgravity [27]. Further analysis of pathways associated with these downregulated genes suggested that the NF-κB pathway was inhibited. Twenty of these 47 genes have promoter regions containing c-REL binding sites, suggesting that the transcription of downstream effectors in the REL/NF-κB pathway may ultimately influence T cell activation in microgravity [27]. Similarly, activation of T cells by various stimulants was inhibited under simulated microgravity using RPM [28]. Expression of the genes that are known for T cell activation were also inhibited [28].

**Figure 1:**
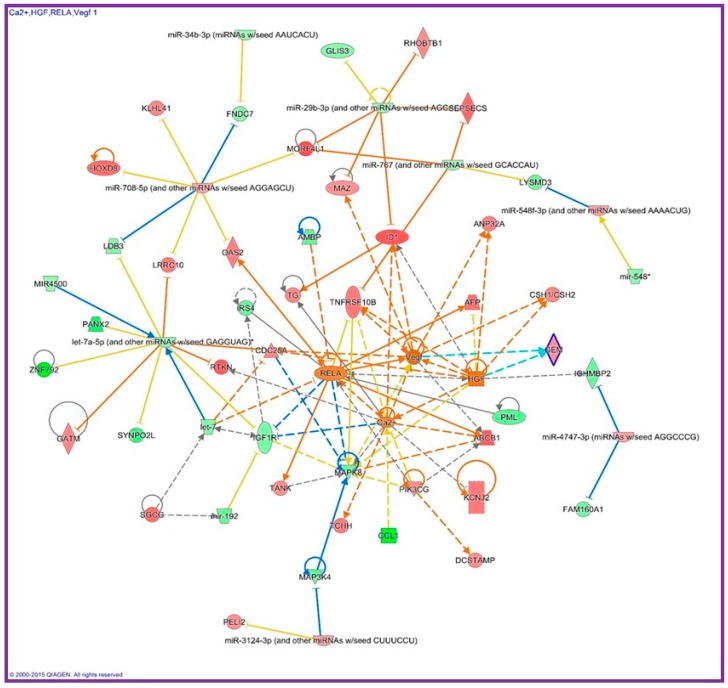
Interaction network identified with genes and miRNAs that were differentially expressed in human fibroblasts after flown on the international space station for 3 days. The chart was generated using Ingenuity Pathway Analysis (Qiagen, Germantown, MD, USA). Activation of NF-κB in space may be responsible for the faster cell proliferation by upregulating *HGF* and *VEGF* [30].

In addition to immune cells, human fibroblasts (AG1522 cells) that were flown to the international space station (ISS) exhibited global miRNA and gene expression profile changes in comparison to the ground controls. Pathway analysis of 29 miRNAs and 170 genes, whose expressions were significantly altered in space, revealed that RELA was one of the top upstream regulators influenced in microgravity [30]. Figure 1 shows the pathway analysis from the genes and miRNAs whose expression was altered after flown in space for 3 days. Activation of the genes in the NF-κB network such as hepatocyte growth factor (*HGF*) and vascular endothelial growth factor (*VEGF*) could be responsible for the observed faster growth rate of these fibroblasts in space. In the gastrocnemius muscle of mice that were flown on the Space Shuttle (STS-108) for 11 days and 19 h, *Nfkbia* mRNA, whose protein IκBα binds to NF-κB in the cytoplasm of the cells, was found to be significantly upregulated than the ground controls [34]. Results of the study suggest that NF-κB may also play a critical role in muscle atrophy in unloading models [34]. Furthermore, thyroid cancer cells cultured on an RPM for 24 h showed lower levels of the NF-κB p65 proteins in comparison to the cells cultured in the 1 g static condition [31].

## 3. Effects of Microgravity on NF-κB in the Immune System

Spaceflight is known to cause immune dysfunction in astronauts, as measured by the redistribution of leukocyte subsets and the reduction of T cell function [6]. Such immune dysfunction can persist for months for the duration of the space missions, and the degree of the changes may depend on the gender of the crewmember [6,48]. Immune dysfunction has also been reported in rodents under HU [49]. For instance, animals under HU showed an impaired ability to respond to the attack of pathogens [50]. The molecular mechanisms by which microgravity induces immune dysfunction have been extensively investigated [51,52,53,54,55]. In addition to microgravity, studies of immune function changes have been conducted using high energy charged particles generated on the ground. Radiation of total doses within the range expected during long-term missions has been shown to induce changes in the immune function [56]. Immune dysfunction has also been reported in animals exposed to a combination of simulated microgravity and high energy protons, as measured by the lack of T cell activation. These animals exposed to these combined stress factors were also susceptible to the non-toxic bacteria *Pseudomonas aeruginosa* [57].

Apart from the role of NF-κB in inhibiting T cell activation, as discussed in Section 2, inhibition of nuclear translocation of NF-κB has been observed as the predominant response to simulated microgravity, in either stimulated or non-stimulated Jurkat cells [29]. Downregulation of *Nfkb1* has also been reported in the spleen of C57BL/6 mice flown on the Space Shuttle for 13 days [35]. A schematic summary of the influence of microgravity on T lymphocyte function including the inhibition of NF-κB nuclear translocation has been recently reviewed [58]. Nuclear translocation of NF-κB under microgravity does not occur in all cell types. For instance, in the rat macrophage cell line NR8383, the translocation of NF-κB to the nucleus was not affected in microgravity [59].

## 4. Effects of Microgravity on NF-κB in Muscle

Muscle atrophy is a major concern for astronauts in microgravity, as the bone and muscle tissues become weak due to prolonged periods of disuse. The role of NF-κB in skeletal muscle atrophy has been widely reported in the literature. NF-κB activation has been found in a number of muscle disorders such as sarcopenia, muscular atrophy, cancer cachexia, inflammatory myopathies, and muscular dystrophies [60]. Studies of sarcopenia in the age-related loss of muscle have reported a four-fold increase in NF-κB protein concentrations in the elderly compared to that of young people [60,61]. In Duchenne muscular dystrophy patients, NF-κB cytokines such as TNF-α were upregulated in their dystrophic muscles [62]. A literature review reveals that skeletal myogenesis requires a delicate balance and timing of both canonical and noncanonical NF-κB signaling pathways, although the mechanisms remain unclear [63].

In a spaceflight-related study, mice were flown to space for 11 days and 19 h, or were subjected to HU on the ground for 12 days, for investigation of gene expressions in the muscle [34]. In both groups, the body weight was significantly decreased in comparison to the weight bearing ground controls. Analysis of mRNA in the gastrocnemius muscle showed an increase of *Nfkbia*/*Iκbα* by 2.28-fold after spaceflight. In another study, researchers separated three strains of mice (wild type (WT), *Nfkb1*^−/−^, and *Bcl3*^−/−^) into two groups, weight bearing and HU [36]. Bcl3 is involved in the regulation of transcriptional activation of NF-κB target genes and inhibits the nuclear translocation of the NF-κB p50 subunit contributing to the regulation of cell proliferation [64]. After 6 days, an 18% decrease in gastrocnemius plus plantaris muscle mass was found in the WT HU mice but little to no difference in the muscles of the *Nfkb1*^−/−^ and *Bcl3*^−/−^ mice. Of the 240 genes that were upregulated in the wild type HU mice, 185 showed no expression changes in the *NF-κB* or *BCL3* deficient mice, indicating that these genes were either direct or indirect targets of p50 or BCL3. These results suggested that by inhibiting activation of the NF-κB or BCL3 pathways, muscle atrophy can be reduced or eliminated [36]. In human subjects under bed rest conditions for 7 days, a loss of an average of 1.6 kg of total body lean mass was reported, with 50% of the muscle loss coming from the legs [32]. After bed rest, the *NF-κB1* gene expression level in the muscle biopsy was increased by 40% [32].

## 5. Effects of Microgravity on NF-κB in Bone

Bone loss is an important issue due to the fact that bone resorption increases in space and there is a lack of mechanical stimulation for bone formation [4]. The loss of bone mineral density (BMD) has been reported to be approximately 1% per month during spaceflight [4]. The role of NF-κB in bone loss and bone disease has been documented in the function of osteoclasts and osteoblasts, skeletal development, and endochondral ossification [65]. Activation of NF-κB has been found to play a role in many bone diseases, including inflammatory arthritis, osteoarthritis, rheumatoid arthritis, and Paget’s disease [66]. When activated, NF-κB causes TNF to downregulate *SOX9* expression, through this pathway chondrocyte differentiation is inhibited, leading to rheumatoid and osteoarthritis [65].

The relationship between bone loss and NF-κB expression in peripheral blood mononuclear cells (PBMC) of the astronauts after spaceflight has been investigated [33]. After 12–16 days space missions, an increased expression of nearly 500% of NF-κB p65 was detected after flight, and the level remained elevated for 14 days after landing. It was suggested that omega-3 fatty acids or eicosapentaenoic acid can be used as a countermeasure for spaceflight-induced bone loss by inhibiting NF-κB activation [33]. Similar to the study of muscle loss, the role of NF-κB in microgravity-induced bone loss has been investigated with mice deficient in *NF-κB1* [37]. After a 2-week HU, the wild type mice had significant trabecular bone loss compared to the *NF-κB1* deficient group. The WT mice also showed a decrease in BMD of 30% compared to *NF-κB1* deficient mice, suggesting that NF-κB deficiency inhibited the reduction due to mechanical unloading in osteoblastic bone formation and enhanced osteoclastic bone resorption [37].

## 6. Effects of Microgravity on NF-κB in Cardiovascular System

Spaceflight has been shown to have effects on many systems in the human body, the cardiovascular system included. Microgravity has been shown to decrease circulating blood, plasma volume, interstitial fluids, and ventricular stroke volume and inhibit erythropoiesis [67]. NF-κB has been linked to various diseases affecting the cardiovascular system, such as atherosclerosis, myocardial ischemia, and heart failure [68]. It has been reported that NF-κB activation can have divergent functions in the cardiovascular system and has been described as either adaptive or maladaptive, depending on the cellular and physiological context and timing of the activation [69]. Some of the maladaptive roles that are played by chronic NF-κB activation in the cardiovascular system are prolonged inflammatory response leading to heart failure and preventing the removal of damaged cardiac cells which can lead to a decrease in cardiac function [69].

The effects of microgravity on NF-κB have been investigated using rat ventricular heart cells (H9c2) incubated in RWV for 3 h either in simulated microgravity or normal gravity [25]. Western blot analysis showed an increase of 23% of NF-κB p65 in the nuclear lysates of the microgravity exposed cells compared to the dynamic control cells. Analysis with ELISA also found an increase in DNA binding of p65 in microgravity exposed cells compared to the dynamic control [25]. It was also shown in rat ventricular heart muscle exposed to simulated microgravity that NF-κB was significantly increased in the nuclear fraction compared to the control [25].

Space radiation can potentially cause cardiovascular diseases. Although limited data with charged particles is available, assessments of nuclear bomb survivors exposed to low-LET radiation have shown that these individuals have an increased frequency of cardiovascular disease, ischemic heart disease, and stroke [70]. A study of arterial biopsies from patients enrolled in radiotherapy and nonradiated patients found persistent inflammation due to NF-κB activation in the radiated patient’s arteries. In addition, the genes related to the NF-κB pathway were dysregulated for years after the initial radiation exposure [70].

## 7. Effects of Microgravity on NF-κB in Brain

The brain is one of the most important organs that undergoes large biological changes under microgravity. It has been reported that the brain experiences tremendous fluid shift during space travel and thereby results in determinant behavioral change in astronauts [71]. Microgravity induces stress in the brain and therefore activates several transcription factors to cope with the stress [72]. Among the transcription factors, NF-κB plays a pivotal role in neuronal cell survival against neurotoxins by inducing genes favoring cell survival [73]. In addition, it is been reported that the NF-κB activity was elevated in chronic neurodegenerative disorders [74].

Simulated microgravity with HU has shown that oxidative stress induced NF-κB activation in different regions of mouse brain [38]. Proteomic analysis of simulated microgravity-induced mouse hypothalamus revealed the imbalance in oxidative stress [75]. Activation of NF-κB in the brain has also been demonstrated in mice exposed to charged particles. After high energy proton irradiation, oxidant and antioxidant levels in the brain of the mice under HU were significantly higher than the gravity loaded controls [75]. In rats exposed to high-LET oxygen ions at doses of 0.1, 0.5, and 1 Gy, increased levels of NF-κB and glial fibrillary acidic protein (GFAP) can still be observed in the brain at 75 days post irradiation for 1 Gy, indicating glial cell activation [76].

## 8. Effects of Space Radiation Exposure on NF-κB

Among the risks associated with space radiation exposure are cancer, central nervous system effects, degenerative tissue damage, and acute radiation syndrome [1]. However, only a few effects of space radiation exposure, such as light flashes experienced on their trip to the Moon and in LEO [77,78], elevated chromosome damage in the lymphocytes after long-duration space station missions [79], and early onset of cataracts [80] have been reported in the astronauts. NF-κB is linked to many of the identified radiation risks for spaceflight, particularly in the promotion of tumors [81]. The NF-κB pathway has been shown to regulate the proliferation of colorectal cancer cells [82]. Mutation of *NF-κB* may also be an early event for malignant transformation of precancerous lesions of speckled leukoplakia [83].

Ionizing radiation induces DNA damage, including double-strand breaks, which trigger the activation of NF-κB through ATM dependent or P53-induced death domain (PIDD) dependent pathways [84]. Activation of NF-κB in cells exposed to radiation has been extensively investigated for low-LET radiation [15]. Here, we will only briefly summarize the studies with high energy charged particles that are relevant to space radiation. For charged particles, the degree of NF-κB activation is apparently dependent on the dose and dose rate, the post irradiation time, and the cell type [39]. A dose dependence in the NF-κB binding activity was observed in human monocytes exposed to high-LET Fe ions at 2 and 4 h post irradiation [40]. At longer time points, the Fe ion-induced binding activity diminished. Similar time-dependent NF-κB activation was reported in Chinese hamster cells after exposure to O ions [43]. Detection of NF-κB for low fluence Ar ions (230 keV/µm) may be possible using human embryonic kidney cells transfected with a reporter gene [41]. Activations of NF-κB in cultured cells last mostly for hours after radiation exposure, but they can persist for weeks in animal studies. In the heart and bone marrow tissue of CBA/CaJ mice exposed to Si ions, activation of NF-κB can last up to 6 months post exposure [46]. These long-term activations of NF-κB were associated with chronic inflammation in the exposed animals [47]. The degree of NF-κB activation is also dependent on the quality of radiation. While C ions (LET = 33 and 73 keV/µm) and X rays had comparable potential to activate NF-κB in human embryonic kidney cells [42], Ar (LET = 272 keV/µm) and Ne (LET = 91 keV/µm) ions produced the highest relative biological effectiveness (RBE) of 8.9 [39]. In normal human fibroblasts exposed to Ar microbeam particles, phosphorylation of NF-κB was also observed in the non-exposed cells as a result of nitric oxide-mediated bystander effects [85]. NF-κB has been demonstrated to play a key role in radiation-induced bystander effects [86], but such studies are beyond the scope of this review.

Activation of NF-kB has also been investigated for high energy protons or protons of mixed energy that simulate SPE. In bone marrow cells isolated from BALB/cJ mice after whole-body exposure to 100 MeV protons at 5 or 10 mGy/min, the dose rate effects have been investigated [44]. At 1.5 h post irradiation of a total dose of 1 Gy, the level of NF-κB activation was significantly higher than the non-exposed controls only in the 10 mGy/min group. On the contrary, the 5 mGy/min group showed a higher level of NF-κB activation than the 10 mGy/min group at 1 month post irradiation [44]. In a study of the effects of SPE on immune functions, C57BL/6 mice were whole-body exposed to protons of mixed energies. At 21 days post exposure, the total and the active forms of NF-κB in T cells isolated from spleens of the mice increased [45]. Such activations were modified with prior exposure to a low dose γ rays. 

Microgravity can potentially influence the cellular response to radiation exposure through the NF-κB pathways, and vice versa. Research aimed at sensitizing tumor cells to radiation treatment has reported that inhibition of NF-κB generally enhances cell killing [87]. On the other hand, activation of NF-κB through low-dose radiation can reduce DNA damage from subsequent radiation exposure at high doses [88]. In space, the NF-κB subunits can be up- or downregulated depending on the cell type. Such alterations can potentially impact the cellular response to DNA damages induced by different stress factors such as radiation and microgravity.

## 9. Conclusions

The harmful space environment is known to cause detrimental health consequences in humans. Different from most other reviews that focus on specific health risks, this review attempts to focus on the NF-κB pathway that is known to play a role in many of the health risks associated with spaceflight. Sufficient evidence has been accumulated to indicate that the NF-κB pathway is altered by either microgravity or space radiation. Such evidence was found in transcriptomics studies that explored all possible pathways, and in experiments targeting specifically NF-κB across different cell types and organ tissues. Whether the NF-κB pathway is activated or inhibited in space is dependent on the cell types. In most cell types, NF-κB is activated in response to different types of stress factors. In immune cells, however, microgravity downregulates the expression of NFKBIA, which corresponds to the inhibition of T-cell activation. In either case, altered NF-κB activities in the space environment appeared to potentially impact health in a negative direction. Investigations of NF-κB in some other organs such as testis have also been reported [89], but this review does not cover all areas.

Potential association of altered NF-κB and spaceflight-related health risks does not necessarily imply causality. More investigations using cells or animals deficient in certain subunits of NF-κB are needed to determine whether manipulation of the NF-κB pathways will reduce the associated risks. Two of the studies using mice deficient in *NF-κB* demonstrated that simulated microgravity induced significantly less bone or muscle loss in comparison to the WT controls [23,36], suggesting that altering NF-κB expressions should be considered as a countermeasure for these risks. In fact, manipulation of NF-κB has been considered for treatment of various diseases [90]. For instance, countermeasures against microgravity-induced bone loss through inhibition of NF-κB have been suggested by using omega-3 fatty acids or eicosapentaemoic acid [33].

**Figure 2:**
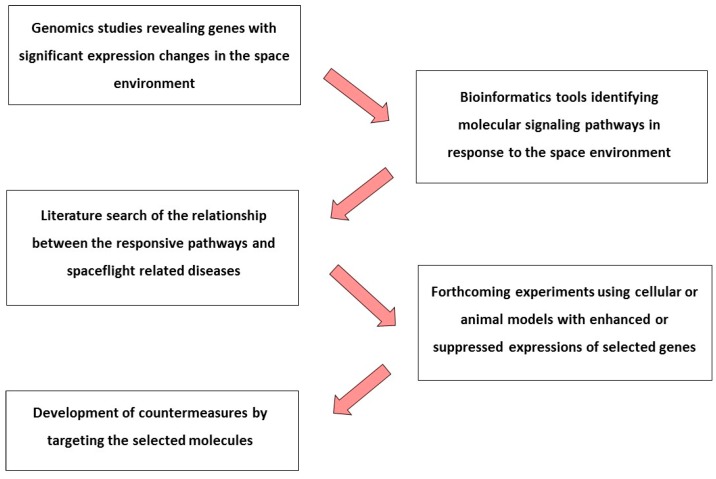
Illustration of the use of omics data from identification of pathways that are responsive to the space environment to the development of countermeasures.

Genomics is used to identify biological pathways and protein networks underlying complex cellular processes. This review of NF-κB provides an example of how omics data could be applied for addressing spaceflight related health risks, as shown in Figure 2. Omics studies might reveal unknown, and perhaps unique, pathways in response to the space environment. The identification of genes affected by the space environment allows researchers to explore potential protein networks and determine cellular pathways associated with spaceflight-related diseases. Key genes involved in the relevant pathways can then be targeted in forthcoming experiments using cells or animals with enhanced or suppressed gene expression. Such genetic manipulations may contribute to the development of countermeasures at the molecular level.

## Figures and Tables

**Table 1:** Summary of studies on NF-κB in cells or animals conducted in space or using simulated microgravity on the ground. RWV, rotating wall vessels; ISS, international space station; RPM, random positioning machine; PBMCs, peripheral blood mononuclear cells; STS, space transportation system; WT, wild type; HU, hindlimb unloading; RT, reverse transcription; PCR, polymerase chain reaction; ELISA, enzyme-linked immunosorbant assay; µCT, micro computed tomography.

Cells/Organism	Type of Microgravity	Type of Analysis	Length of Exposure	Result	Author
**Cell Line**					
Rat cardiac cells (H9c2)	RWV	Western blot; ELISA on nuclear lysates	3 h	Increased NF-κB p65 DNA binding activity	Kwon et al. [25]
Human lymphoblastoid cells (TK6)	RWV	Microarray; PCR array on whole cell lysates	72 h	Differential expression profile of genes and miRNAs identifying activation of the NF-κB pathway	Mangala et al. [26]
Activated human T cells	ISS	Microarray on whole cell lysates	1.5 h	Suppressed expression of cREL/NF-κB gene targets	Chang et al. [27]
Activated human T cells	RPM	RT-PCR on whole cell lysates	4 h	Suppressed expression of NF-κB gene targets	Boonyaratnakornkit et al. [28]
Human Jurkat T cells	RWV	Western blot on nuclear lysates	5 min	Decreased translocation of NF-κB p65 protein	Paulsen et al. [29]
Human fibroblasts (AG1522)	ISS	Microarray; PCR array on whole cell lysates	72 h	Differential expression profile of genes and miRNAs identifying activation of the NF-κB pathway	Zhang et al. [30]
Human Thyroid cancer cells (FTC-133)	RPM	Microarray; Western blot on whole cell lysates	24 h	Increased NF-κB p65 protein level	Grosse et al. [31]
**Human Study/Mouse Cells**					
Human muscle tissue	Bed-rest	RT-PCR; Western blot on tissue sample	7 days	Increased expression of *NFKB1*, No change in total and phosphorylated NF-κB p65 protein level	Drummond et al. [32]
Human PBMCs; Murine monocyte/macrophage cells (RAW264.7)	RWV for RAW264.7 cells; Space Shuttle for PBMCs	Western blot and ELISA on nuclear lysates for RAW264.7 cells; Immunocytochemical method for PBMCs	24 h for RAW264.7 cells; 12–16 days for PBMCs	Increased NF-κB p65 DNA binding activity and increased p65 protein level in RAW264.7 cells; Increased NF-κB p65 protein level after spaceflight; Omega-3 fatty acids or eicosapentaenoic acid reduced NF-κB p65 protein level	Zwart et al. [33]
**Mouse Study**					
Mouse Gastrocnemius (C57BL/6)	STS-108; HU	Microarray; RT-PCR on tissue sample	11 days 19 h	Increased expression of *Nfkbia/Iκbα*	Allen et al. [34]
Moue spleen (C57BL/6)	STS-135	PCR array on tissue sample	13 days	Suppressed expression of *Nfκb1*	Gridley et al. [35]
WT and *Nfkb1*^−/−^ or *Bcl-3*^−/−^ mouse gastrocnemius and plantaris muscles (B6129PF2/)	HU	Microarray; RT-PCR on tissue sample	10 days	Reduced muscle atrophy in *Nfkb1*^−/−^ or *Bcl-3*^−/−^ mice; Increased expression of NF-κB gene targets in WT mice	Wu et al. [36]
WT and *Nfkb1*^−/−^ mouse bone (C57BL/6)	HU	µCT on tibias and femurs bone mass; Western blot on tissue sample	2 weeks	Reduced bone loss in *Nfkb1*^−/−^ mice in comparison to WT, Increased NF-κB p50 protein level in WT HU group	Nakamura et al. [37]
Mouse brain (BALB/c)	HU	EMSA on nuclear lysates	7 days	Increased NF-κB DNA binding activity	Wise et al. [38]

**Table 2:** Summary of studies on NF-κB in cells or animals exposed to charged particles. SPE, solar particle events; RBE, relative biological effectiveness; EMSA, electrophoretic mobility shift assay; Gy, gray.

Cells/Organism	Radiation Type and Quality	Dose/Dose Rate Range	Method	Result	Reference
**Cell Line**					
Human embryonic kidney cells (HEK 293)	C (34 keV/µm) Ne (91 keV/µm); Ar (272 keV/µm); Ni (906 keV/µm); Pb (9674 keV/µm)	0.1–60 Gy ~1 Gy/min	Fluorescence d2EGFP reporter gene assay	RBE for NF-κB dependent *d2EGFP* expression varies by particle types with peak RBE = 8.9	Hellweg et al. [39]
Human monocytes (MM6)	Fe	0.2–1.4 Gy 1 Gy/min	EMSA on nuclear lysates	NF-κB DNA binding activity increased in a dose and time dependent manner	Natarajan et al. [40]
Human embryonic kidney cells (HEK 293)	Ar (230 keV/µm)	0.2–30 Gy	Fluorescence d2EGFP reporter gene assay	Increased NF-κB dependent *d2EGFP* expression in a dose and time dependent manner	Baumstark-Khan et al. [41]
Human embryonic kidney cells (HEK 293)	C (33 and 73 keV/µm)	0.2–20 Gy ~1 Gy/min	Fluorescence d2EGFP reporter gene assay	NF-κB dependent *d2EGFP* expression was comparable to X-rays	Hellweg et al. [42]
Chinese hamster cells (V79)	O	1 Gy	Western blot on whole cell lysates	Decreased NF-κB p65 level at 30 min post irradiation, but the level recovered at longer time points	Mitra et al. [43]
**Mouse Study**					
Mouse bone marrow (BALB/cJ)	Proton (0.7 keV/µm)	1 Gy 5 and 10 mGy/min	ELISA on nuclear lysates	NF-κB p65 DNA binding activity was both dose rate and time dependent	Rithidech et al. [44]
Mouse spleen T cells (C57BL/6)	SPE protons	1.7 Gy delivered over 36 h	ELISA on whole cell lysates	Increased total and phosphorylated form of NF-κB p65	Rizvi et. al. [45]
Mouse heart and bone marrow (CBA/CaJ)	Si (77 keV/µm)	0.1–0.5 Gy 10 mGy/min in two fractionations with 15 days apart	ELISA on nuclear lysates	Increased NF-κB p65 DNA binding activity up to 6 months post irradiation	Tungjai et al. [46]
Mouse liver (CBA/CaJ)	Ti (107 keV/µm)	0.1–0.5 Gy 0.01 Gy/min	ELISA on nuclear lysates	Increased level of NF-κB p65 DNA binding activity up to 6 months post irradiation	Jangiam et al. [47]
